# Shelf Auditing Based on Image Classification Using Semi-Supervised Deep Learning to Increase On-Shelf Availability in Grocery Stores

**DOI:** 10.3390/s21020327

**Published:** 2021-01-06

**Authors:** Ramiz Yilmazer, Derya Birant

**Affiliations:** 1 Graduate School of Natural and Applied Sciences, Dokuz Eylul University, Izmir 35390, Turkey; ramiz.yilmazer@ceng.deu.edu.tr; 2 Department of Computer Engineering, Dokuz Eylul University, Izmir 35390, Turkey

**Keywords:** on-shelf availability, semi-supervised learning, deep learning, image classification, machine learning, explainable artificial intelligence

## Abstract

Providing high on-shelf availability (OSA) is a key factor to increase profits in grocery stores. Recently, there has been growing interest in computer vision approaches to monitor OSA. However, the largest and well-known computer vision datasets do not provide annotation for store products, and therefore, a huge effort is needed to manually label products on images. To tackle the annotation problem, this paper proposes a new method that combines two concepts “semi-supervised learning” and “on-shelf availability” (SOSA) for the first time. Moreover, it is the first time that “You Only Look Once” (YOLOv4) deep learning architecture is used to monitor OSA. Furthermore, this paper provides the first demonstration of explainable artificial intelligence (XAI) on OSA. It presents a new software application, called SOSA XAI, with its capabilities and advantages. In the experimental studies, the effectiveness of the proposed SOSA method was verified on image datasets, with different ratios of labeled samples varying from 20% to 80%. The experimental results show that the proposed approach outperforms the existing approaches (RetinaNet and YOLOv3) in terms of accuracy.

## 1. Introduction

Machine learning techniques have been applied to different areas in the retail sector. One of them is the monitoring on-shelf availability (OSA) in grocery stores. Providing high OSA is a key factor to increase profits. When a product that a shopper looks for is not available on its designed shelf, also known as “out-of-stock” (OOS), this causes a negative impact on customer behaviors in the future. According to the research reported by Corsten and Gruen [1], when a product is not available on the designed shelf space, 31% of consumers buy the product from a different store, 26% of them buy a different brand, 19% of them buy a different size of the same brand, 15% of them buy the same product at a later time, and 9% of them buy nothing. Besides, another study [2] showed that the “out of the stocks” rate is about 8% in the United States and Europe. For this reason, OSA has a significant effect on business profit. The remaining products can be checked using an inventory management system, but it only shows the number of products in the stock. These products, available in stock, might not be on the shelves. Current inventory systems cannot understand the number of products on the shelves. OSA is checked by employees manually at most of the grocery stores. This approach is not effective and sustainable since it continuously requires human effort.

There are several studies to automate monitoring OSA. These studies consider the subject from different perspectives. One of them proposes radio frequency identification (RFID) tagging to monitor product quantity on the shelves [3], but this approach is not cost-effective to implement the technology and integrate it into existing systems [4]. Some of them applied traditional image processing techniques to detect the presence and absence of the product, such as [5], and some of them used deep learning approaches for object detection on the shelves, such as [6]. When all these previous works are examined, there are pros and cons of these approaches. When traditional image processing methods have been used, such as histogram of oriented gradient (HoG) for feature extraction and support vector machine (SVM) for a classifier, they have limited performance even if on the large datasets and their performances are hard to be increased. In addition, the visual similarity among the different products of the same brand can lead to misclassification. On the other hand, when deep learning (DL) approaches were used such as recurrent convolutional neural network (RCNN) or you only look once (YOLO), high accuracies could be achieved. The DL algorithms require annotated images to train, but the largest and well-known computer vision datasets do not provide annotation for store products, and therefore a huge effort is needed to manually label products on images.

A semi-supervised learning approach can produce results with satisfactory accuracy using an amount of labeled data and a much larger amount of unlabeled data in the training phase [7]. This paper proposes a new method that combines two concepts “semi-supervised learning” and “on-shelf availability” (SOSA) for the first time. An important advantage of the proposed SOSA method is that it solves the OSA problems where labeled image data is scarce. Labeling OSA image data is an expensive, tedious, difficult, or time-consuming process since it requires human labor. The proposed SOSA method deals with the design of on-shelf availability monitoring models in the presence of both labeled and unlabeled image data.

From another perspective, users have to understand and trust the constructed OSA model as a kind of artificial intelligence (AI) application. Besides, if their requirements change, these applications have to be managed according to their needs [8]. Our paper proposes the first demonstration of explainable artificial intelligence (XAI) on OSA.

The main contributions and novelty of this paper can be listed as follows. (1) This is the first study that combines two concepts “semi-supervised learning” and “on-shelf availability” (SOSA) for the first time. (2) It is the first time that YOLOv4 deep learning architecture is used for exploring OSA. (3) This study is also original in that it compares three deep learning approaches for monitoring OSA in the retail sector. (4) To the best of our knowledge, this is the first demonstration of explainable AI on OSA. This paper also presents a new software application, called SOSA XAI, with its capabilities and advantages.

In the experimental studies, the effectiveness of the proposed SOSA method was verified on image datasets, with different ratios of labeled samples varying from 20% to 80%. We focused on category-based detection of empty and almost empty shelves. For this purpose, we used both labeled and unlabeled images that include 49,573 products grouped under three categories. The experimental results show that the proposed approach outperforms the existing approaches (RetinaNet and YOLOv3) in terms of accuracy.

The remainder of this article is structured as follows: Section 2 summarizes the related works on OSA. In Section 3, we first briefly introduce background information of the compared algorithms and then explain semi-supervised learning of the OSA (SOSA) concept with its definitions. This section also describes our demonstration of XAI on OSA. Section 4 presents the experimental results and Section 5 presents discussions about the obtained results. Finally, concluding remarks are presented in Section 6.

## 2. Related Works

In the literature, researchers have approached the subject from different perspectives. They proposed both technical and managerial solutions to minimize OOS and monitor OSA. Different technical solutions have been proposed, such as analyzing textual data on the relational database management systems (RDMS) and applying computer vision-based techniques. In this paper, we focus on computer vision-based techniques, and previous works were examined based on this scope.

Moorthy et al. [5] applied image processing techniques to detect the presence and absence of the product in front of the shelf using MATLAB. In addition, they worked on the positioning of products in retail stores. The feature extraction technique was used for object detection and a speeded up robust features (SURF) algorithm was used for this purpose. Reference images were given to the system and target images were taken from video or camera devices as shelf images. Some pre-process operations were applied to images before comparison of input and target images and extracting features using the SURF algorithm. At the end of these steps, the product was evaluated for missing or misplaced. Besides, they [9] proposed the image processing approach to provide high OSA in retail. They collected images from shelves to use reference images. Reference images and target images were compared using image processing techniques. When comparison showed missing products on the shelf, the system sent messages to the manager or responsible person in the store. A similar template matching approach was used to develop OSA monitoring software in another study [10]. Moreover, some researchers [11] worked on high-resolution panoramic images and proposed a supervised learning approach. Panoramic images were created using a wide-angle fisheye camera and an accelerated-KAZE (AKAZE) feature detector was applied. Labels were detected from the panoramic images of shelves. A cascade classifier approach was used for this purpose.

Kejriwal et al. [12] worked on counting products from shelf images using robot-based equipment. Several cameras were placed on either side of the robot. The robot was moved between shelves and video data were collected from shelves. Several methods were applied to these collected datasets. The first method was used to recognize the product and the second one was used to count the product. A k-d tree was created and the SURF approach was used to recognize the product by using the nearest neighbor search. Two different techniques were used for product count. The first one is the product counting method in which repeating features were counted to understand the number of products on the image by using the SURF method. Secondly, rectangular bounding boxes were drawn around the products and these boxes were counted to understand the number of products by using the random sample consensus (RANSAC) method. Moreover, the grid search approach was applied in the neighborhood of each found product on the image. Some undetected products were tried to find in this way.

Higa et al. [6] studied product changes on the shelf. They focused on taken or returned products on the shelf. Videos, captured from a surveillance camera, were used for the study. Low-quality videos, 480 × 270 pixels, and 1 fps were used to eliminate storage problems but another problem occurred at this time. Moving objects was difficult to track from low-resolution videos. They used background subtraction and the convolutional neural network (CNN) approach to detect changes of products on the shelf. CNN was used based on CaffeNet and four classes were determined for CNN. The extended version of the study [13] proposed analyzing consecutive images when the customers stood in front of the shelf. The Hungarian method was used to analyze consecutive images. Moreover, the Canadian Institute for Advanced Research (CIFAR)-10-based network and CaffeNet-based network were used to detect change regions. In addition, a heatmap was generated to show the customer’s behavior using captured images. Frequently accessed shelves were analyzed with this approach.

Some studies exist about planogram compliance checking. Planograms are created to standardize placed products on shelves for chain supermarkets. Another aim of creating a planogram is that, providing the best customer experience for promotions. Liu et al. [14] studied automatic planogram checking compliance using recurring patterns. These planograms are created by headquarters and sent to store managers. Store managers are responsible for applying planograms to store shelves. In a conventional way, they try to check planogram compliance manually. The proposed automatic checking compliance was done without using template images. Planogram was taken as XML format and parsed. Some matrix operations were applied to detect recurring patterns. The extended version of the study [15] focused on spectral graph matching and speed improvements using a divide-and-conquer approach. Besides, Saran et al. [16] studied visual analysis for planogram compliance. They compared the reference template image to the target image. Their study focused on the presence of products, placements of products, and product count. The Hausdorff map approach was used for the presence of products. This presence of products was a group under two classes which were complete and partial cases. The Euclidean distance approach was used to identify completely missing cases. A binary distance map approach was used with the self Hausdorff map. Shelf rows were identified using the Sobel derivatives extractor and Hough line extractor. The Sobel derivatives extractor was used for vertical changes and the Hough lines extractor was used for horizontal changes. Finally, texture features and color features were used to count products from images. The color feature was used to eliminate false positives.

Briefly, the summarized related works above focus on OSA monitoring and planogram checking based on traditional image processing and deep learning techniques using different approaches. Image processing techniques have performance issues for huge datasets. Moreover, for template matching, reference images have to be stored in a database and this is not suitable for real-world applications. On the other hand, labeled images are needed using deep learning techniques for object detection on the shelves. The largest and well-known computer vision datasets do not provide labeled images for store products. This process is time-consuming and quite expensive since it requires human labor costs. This paper proposes a new method that combines two concepts “semi-supervised learning” and “on-shelf availability” (SOSA) for the first time. The main advantage of the proposed SOSA method is that satisfactory results can be achieved using a small amount of labeled data and a large amount of unlabeled data for OSA monitoring.

Monitoring on-shelf availability has very little coverage in the literature, only a few numbers of detailed analyses [5,6,9,10,11,12,13,16] have been performed. Table 1 shows the comparison of our study with the previous studies aforementioned. Our approach differs from the existing approaches in many respects and has numerous advantages over the rest as follows:

- First, some of the previous studies [5,9,10,11,12,16] used traditional techniques such as image processing (IP) to monitor OSA, whereas we used deep learning techniques. In IP-based approaches, a huge amount of reference images has to be stored to match the target image and for every product updating, reference images have to be updated manually. In this context, an important advantage of our method is that it does not require any reference image and therefore it does not need manual updating when products are updated. Moreover, it automatically extracts features from an input image thanks to deep learning.
- Second, the previous deep learning-based studies used different network structures such as the CaffeNet-based network [6,13] and CIFAR-10-based network [6,13], whereas we designed a novel network architecture that consists of RetinaNet, YOLOv3, and YOLOv4 detectors. Here, the advantage of our approach is that it builds three different models and selects the best one, and hence, satisfactory results can be achieved by the selection of the best model.
- Third, the previous deep learning-based studies used two-stage detectors. On the other hand, in this study, we benefit from one-stage detectors because of their speed and achieving satisfactory accuracy results for OSA monitoring.
- Four, our study differs from the rest in that we adapted the semi-supervised learning concept, and therefore we benefited from both labeled and unlabeled data. Here, the main advantage is that our method reduces the need for labeling images which is an expensive, tedious, difficult, and time-consuming process since it requires human labor. Satisfactory results can be achieved using a small number of labeled images. Moreover, the proposed method will expand the application field of machine learning in grocery stores since a large amount of OSA data generated in real-life are unlabeled.
- Finally, differently from the previous studies, we introduced an explainable AI concept into OSA. The developed new SOSA XAI software application allows users to manage, understand, and trust the model when monitoring OSA.

## 3. Materials and Methods

### 3.1. Deep Learning for Object Detection

One of the main tasks of computer vision is object detection. It deals with detecting objects along with their locations and classes (such as cars, fruits, food products) in images. Object detection methods can be grouped into two categories: traditional image processing methods and deep learning-based detection methods. Recently, deep learning-based object detection methods have become popular because of outstanding results. In addition, deep learning-based object detection methods can be divided into two categories as two-stage detectors such as regions with convolutional neural networks (R-CNN) [17], faster R-CNN [18], feature pyramid network (FPN) [19], and one-stage detectors such as RetinaNet [20], YOLOv3 [21], and YOLOv4 [22]. In the general structure of two-stage detectors, feature extraction is applied to the input image and generated proposed regions using different methods in the first stage. From these proposed regions, the locations (bounding boxes) and classes of the objects are determined in the second stage. In contrast to two-stage detectors, the region proposal stage is skipped and directly learns class probabilities and bounding box locations from the input image like a simple regression problem. In this study, we decided to work on one-stage detectors because of their speed and achieving satisfactory accuracy results for OSA monitoring.

**Figure 1:**
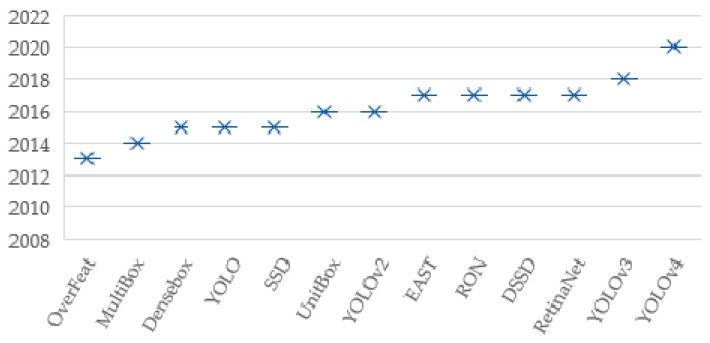
History of one-stage detectors.

The history of one-stage detectors is shown in Figure 1. Since 2012, CNN [23] has been used to be able to learn from features of images robustly. Before this year, traditional detection methods such as image processing were used for object detection tasks. After this year, computer vision techniques have improved very rapidly and novel deep learning-based detection methods have been proposed. The existing detector methods can be grouped under two categories: methods with one-stage detector and methods with two-stage detectors. The first one-stage detector method was proposed in 2013, and since then, at least one novel approach has been proposed each year. Each approach solved the object detection problem from a different perspective to increase accuracy, and each one achieved higher accuracy results from previous ones. For this reason, three of the latest published one-stage detectors (RetinaNet, YOLOv3, YOLOv4) have been selected for this study.

Lin et al. [20] proposed the RetinaNet one-stage object detection model. It uses a new focal loss function to handle class imbalance issues more effectively according to alternative previous approaches. Their model has one backbone network and two subnetworks. Feature map is computed in the backbone network and the output of this network is used as input for two subnetworks. Bounding boxes and classifications are found separately in each subnetwork. FPN is used as a backbone network for RetinaNet. Their proposed architecture improved the performance of the model compared to standard top-down CNN architecture.

YOLOv3 is a one-stage detector that combines FPN, CNN, and the non-maximum suppression algorithm [21]. CNN is used for the feature extraction process. In addition, it has been integrated with the Darknet-19 and residual neural network (ResNet) network structures for feature extraction. On the other hand, a shortcut connection is added to the model. Fifty-three convolution layers are existing with dropout and batch normalization operations in the feature extraction network. The network is used as a backbone network and named Darknet-53. YOLO works on the entire image during the train and test processes.

YOLOv4 is an improved version of previous YOLO models. It combines the YOLOv3 head and path-aggregation network (PANet) for detection steps [22]. PANet is used instead of FPN in the model. The novel backbone, called cross stage partial Darknet-53 (CSPDarknet-53), is used. Some blocks are added such as spatial pyramid pooling (SPP) to increase the receptive fields on the backbone. Besides, YOLOv4 provides data augmentation to expand the training dataset to improve the accuracy of the network without extra inference time. The Mosaic data augmentation method that combines four images in the training phase was proposed.

### 3.2. Proposed Approach: Semi-Supervised Learning on OSA (SOSA)

This paper proposes a new approach: semi-supervised learning on OSA (SOSA). The proposed method combines two concepts “semi-supervised learning” and “on-shelf availability” for the first time to decrease OOS by automatically detecting and classifying products using shelf images. For this purpose, SOSA builds a classification model that allows the detection of “Product”, “Empty Shelf”, and “Almost Empty Shelf” classes by using both labeled and unlabeled image data. The aim of the SOSA approach is section-based detection of empty and almost empty shelves based on the semi-supervised learning principle.

#### 3.2.1. The General Structure of the Proposed SOSA Method

**Figure 2:**
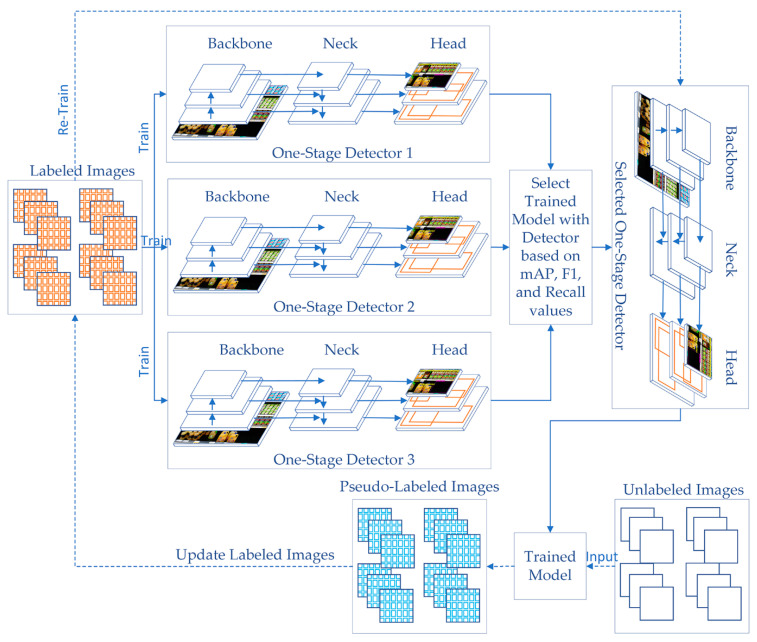
The general structure of the proposed SOSA approach.

Figure 2 shows the general structure of the proposed SOSA approach. SOSA consists of the following main steps. The first step is to train three one-stage detectors on the existing labeled image data. After that, the best-trained model is selected using the evaluation metrics such as mean average precision (mAP), F1-score, and recall measures. In the next step, the unlabeled image data is labeled by the constructed classifier via their predictions, which is commonly referred to as pseudo-labeled data. Lastly, the final classifier is built by using both originally labeled and pseudo-labeled image data. Hence, SOSA allows unlabeled image data to be introduced to the training process in an efficient manner.

In the preprocessing phase of the SOSA method, the images, which are taken from different perspectives in front of the shelves, are labeled to use for the first training. Images are labeled according to the product type(s) it includes. Besides, empty and almost empty shelves are labeled on the images. If one or two of the related products remained on the shelf, these products’ areas are labeled as “Almost Empty”.

#### 3.2.2. The Formal Definition of the Proposed SOSA Method

Assume that labeled dataset *D* = {(*x*_1_, *y*_1_), (*x*_2_, *y*_2_), …, (*x_n_,* *y_n_*)} has *n* images with labeled products. Each component (*x, y*) is composed of *d*-dimensional vector (*x*) from a given input space *X*, such that *x* ∈ *X*, and the output variable (*y*), where *y* ∈ *Y* = {*c*_1_, *c*_2_, …, *c_m_*} has *m* class labels. Unlabeled dataset *U* = {*x_n_*_+1_, *x_n_*_+2_, …, *x_n_*_+_*_s_*} has *s* unlabeled images. We are especially interested in OSA images where labeling the images is difficult and expensive since it requires human labor. The SOSA method considers both *D* and *U* to find a decision function *f*: *X* ↦*Y* that can correctly predict the class labels *ŷ* of a given unseen input sample image *SI*. *Z* refers to one-stage detectors and *Z* = {*z*_1_, *z*_2_, …, *z_k_*} has *k* detectors. In this study, three one-stage detectors are used, where *z*_1_ refers to RetinaNet, *z*_2_ refers to YOLOv3, and *z*_3_ refers to YOLOv4, and so *k* is set to 3.

**Definition** **1.** *Semi-supervised learning on on-shelf availability* (SOSA) refers to a machine learning approach that builds a model to correctly detect “Product”, “Empty Shelf”, and “Almost Empty Shelf” regions from an input image by using both labeled and unlabeled image data, which are taken from different perspectives in front of the shelves.

Algorithm 1 gives the general framework of the proposed SOSA approach. The algorithm consists of five steps. In the first step, labeled dataset *D* is split as *D_Train_*, and *D_Test_* based on the given percentage. In the first loop, a model *z_c_* is trained for each one-stage detector. Trained models are added to *Z*. In the second step, the constructed models are tested using labeled test dataset *D_Test_* and obtained prediction results are compared to select the best one-stage detector that has maximum success rates. The selected-one stage detector is assigned to *SD*. In the third step, a query instance *x_i_* ∈ *U* is submitted to the selected detector *SD*, and its estimation *ŷ* is assigned to *x_i_* as a pseudo-label. This process is repeated for each instance in the unlabeled dataset *U* to generate all pseudo labels. At the end of this labeling operation, labeled dataset *D* is augmented with pseudo-labeled images. In the next step, the classifier is re-trained by using the new dataset *D* which contains both labeled and pseudo-labeled images. Finally, a sample image *SI* is given to the trained model *TM* to be classified, and the predicted classes *ŷ* are obtained from the algorithm.
**Algorithm 1** SOSA: Semi-Supervised Learning on OSA**Inputs:**  *D*: Labeled dataset *D* = {(*x*_1_, *y*_1_), (*x*_2_, *y*_2_), …, (*x_n_*, *y_n_*)} with *n* instances
      *U*: Unlabeled dataset *U* = {*x_n_*_+1_, *x_n_*_+2_, …, *x_n_*_+_*_s_*} with *s* instances
      *Z*: One-stage detectors *Z* = {*z*_1_, *z*_2_, …, *z_k_*} with *k* detectors
      *Y*: Class labels *Y* = {*c*_1_, *c*_2_, …, *c_m_*} with *m* classes
      *SI*: Sample image
**Outputs:** *TM*: Trained model
      *ŷ*: Predicted class labels for the products included in the sample image
**Begin:**
  *D_Train_* = *Split*(*D*, *n* * *percentage*)
  *D_Test_* = *Split*(*D*, (*n* − (*n* * *percentage*)))
  //Step 1—Training with labeled data
  **for**
*c* = 1 **to**
*k*
**do**
    **foreach** epoch
      **foreach** (*x_i_*, *y_i_*) **in**
*D_Train_*
        *z_c_* = Train(*x_i_*, *y_i_*)
      **end foreach**
    **end foreach**
    *Z* = *Z* ∪ *z_c_*
  **end for**
  //Step 2—Testing one-stage detectors and selecting the best one
  **for**
*c* = 1 **to**
*k*
**do**
    **foreach** (*x_i_*, *y_i_*) in *D_Test_*
      *Prediction* = *z_c_*(*x_i_*)
      *PredictionResult_c_* = *PredictionResult_c_* ∪ *Prediction*
    **end foreach**
  **end for**
  *SD* = *MAX* (*PredictionResult_c_*)  //*SD*: Selected detector
  //Step 3—Labeling unlabeled image data and generating pseudo-labels
  **foreach**
*x_i_*
**in**
*U*
    *ŷ* = *SD*(*x_i_*)
    *D*.*Add*(*x_i_*, *ŷ*)
  **end foreach**
  //Step 4—Re-training the model with pseudo-labeled data  *TM* = *Train*(*D*)
  //Step 5—Classifying a sample image
  *ŷ* = *TM*(*SI*)
**End Algorithm**

After the preprocessing phase, the algorithm splits the labeled data into training and test sets according to a given ratio. For each one-stage detector, a model is built by using the training set. At the end of the training phase, three trained models are tested using the test set. As test results, the average precision (*AP*), mean average precision (*mAP*), F1-score, and recall values are calculated based on intersection over union (IoU) threshold value. IoU computes the intersection over the union of the given bounding box and the predicted bounding box. The formulas are given in Equations (1) to (5):

(1) $$\mathrm{Precision}=\frac{\mathrm{True}\;\mathrm{Positive}\;(\mathrm{TP})}{\mathrm{True}\;\mathrm{Positive}\;\left(\mathrm{TP}\right)+\mathrm{False}\;\mathrm{Positive}\;(\mathrm{FP})},$$

(2) $$\mathrm{Recall}=\frac{\mathrm{True}\;\mathrm{Positive}\;(\mathrm{TP})}{\mathrm{True}\;\mathrm{Positive}\;\left(\mathrm{TP}\right)+\mathrm{False}\;\mathrm{Negative}\;(\mathrm{FN})},$$

(3) $$F1\mathrm{Score}\;=\;2\cdot \frac{\mathrm{Precision}\cdot \mathrm{Recall}}{\mathrm{Precision}+\mathrm{Recall}},$$

(4) $$\mathrm{AP}=\frac{1}{N}\cdot \overset{N}{\underset{i=0}{\sum }}{\mathrm{Precision}}_{i},$$

(5) $$\mathrm{mAP}=\frac{1}{\left|T\right|}\cdot \underset{t\in T}{\sum }{\mathrm{AP}}_{t},$$

where |*T*| is total of all *AP*s calculated for each class and *N* is the number of instances in the test set.

After the testing phase, the obtained test results are compared with each other and the detector that has the highest accuracy is selected as the final representative model. In the following phase, semi-supervised learning is performed by labeling the unlabeled data using the trained model of the selected detector. After the labeling operation is completed, the pseudo-labeled data is combined with the labeled data, and then the training process is restarted on the selected detector using the extended dataset. At the end of the re-training operation, the newly trained model is saved for further use for the detection of empty and almost shelves based on sections. To understand which section has empty or almost empty shelves, the relative frequency (*RF*) formula is used as given in Equation (6):

(6) $$\mathrm{RF}=\frac{\mathrm{Frequency}\;\mathrm{of}\;\mathrm{One}\;\mathrm{Product}\;\mathrm{Class}}{\mathrm{Total}\;\mathrm{Frequencies}\;\mathrm{of}\;\mathrm{All}\;\mathrm{Products}’\;\mathrm{Classes}}.$$

Each product is labeled at the end of the detection phase on the shelf image and these labels denote the section of products. For each section, *RF* is calculated and the highest *RF* value gives the final section info. For instance, if the highest *RF* value is obtained for the breakfast products, the section is recognized as a breakfast product. Besides, if three empty and two almost empty shelves are detected on the image, the SOSA algorithm gives the following information: “3 empty and 2 almost empty shelves are existing on the breakfast section.”

#### 3.2.3. The Advantages of the Proposed SOSA Method

The proposed method (SOSA) has a number of advantages that can be summarized as follows:

- The traditional OSA applications are limited to using only labeled image data to build a model. However, labeling shelf images are a time-consuming, tedious, expensive, and difficult job because of existing so many products on a one-shelf image, and for this reason, so many human laborers are needed. This is especially true for the OSA applications that include learning from a large number of class labels and distinguishing similar classes. The main advantage of the SOSA method is that it solves OSA problems using a small number of labeled shelf images. The existing labeled dataset is extended by using unlabeled data with automatically assigned labels, and hence, high accuracy results are taken with the SOSA approach in an efficient way.
- Another advantage is that it includes three different one-stage detector models and the model with the highest accuracy is selected at the beginning of the semi-supervised learning. Hence, satisfactory results can be achieved by the selection of the best model.
- The SOSA approach uses three different deep learning techniques (RetinaNet, YOLOv3, and YOLOv4) without any modification or development of the methods. Therefore, SOSA has advantages in terms of easy implementation. It is possible to implement it in Python by using open-source codes available in the related machine learning libraries.
- The main idea behind the SOSA method is to take advantage of a huge amount of unlabeled image data when building a classifier. In addition to labeled data, the SOSA method also exploits unlabeled data to improve classification performance. Thanks to the SOSA method, the unlabeled data instances provide additional knowledge, and they can be successfully used to improve the generalization ability of the learning system.
- Another advantage is that the SOSA method can be applied to any OSA image data without any prior information about the given dataset. It does not make any specific assumptions for the given data.
- Since a large amount of OSA data generated in real-life is unlabeled, the SOSA method will expand the application field of machine learning in grocery stores.

### 3.3. Explainable AI for SOSA

Explainable artificial intelligence (XAI) is a growing research field in recent years. The aim of XAI is to make machine learning and AI applications more understandable to users who are not experts in these fields. From rule-based systems to deep learning systems, the transparency of systems is decreasing. Especially, it is hard to understand and interpret the outputs of deep learning applications by users. Therefore, users see this kind of AI application as a black box. They think that inputs are given to these applications; afterward, something happens magically inside the box, and outputs are generated by AI applications [24]. On the other hand, AI-based applications are increasingly being adopted by different sectors, including retail. Therefore, business stakeholders and users of AI systems should be able to understand their systems to trust outputs and manage these applications to their needs without getting help from AI experts or engineers [25].

There are different concepts of XAI to provide transparency for AI models in the literature. This transparency can be provided for different parts of the model based on requirements. One of them is post-hoc explainability. The purpose of post-hoc is to explain decisions of AI models using different approaches such as text explanation, visualization, explanation by example [8]. The results of the model can be explained using text definitions, graphics, and images for users in this way. Besides, the dataset can be interpreted during the training phase and the dataset can be extended for better accuracy without the need for AI experts.

In this study, we present the first demonstration of XAI on OSA using post-hoc techniques, and therefore, users can understand and trust the constructed OSA AI model in this way. Moreover, they can interpret the detection results of the model and enhance the dataset in the training phase to adapt the application for changes. In this study, for the OSA XAI demonstration, we used the following post-hoc techniques: text explanations, visualizations, and explanations by example.

**Figure 3:**
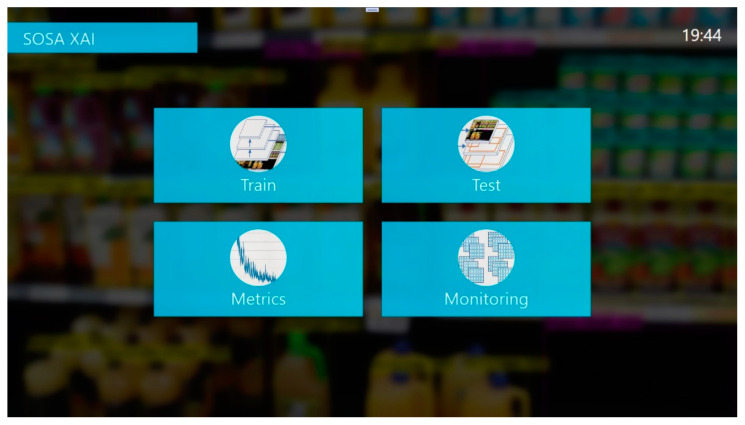
The main screen of SOSA XAI.

**Figure 4:**
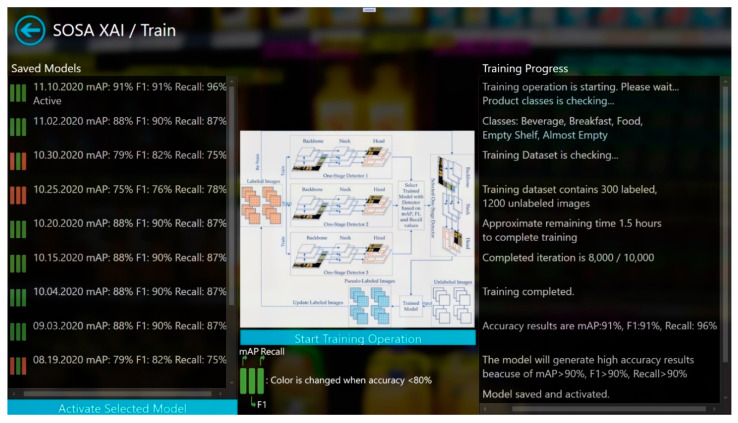
Train screen of SOSA XAI.

In this study, a new software application, called SOSA XAI, was developed to provide understandability for the users. SOSA XAI consists of four main screens. These are “Train”, “Test”, “Monitoring”, and “Metrics” screens. The main screen is shown in Figure 3. Our proposed SOSA method can be managed using the application. After the first initialization, users can re-train the model using the training screen as shown in Figure 4. In the training screen, the creation dates of the models and the obtained results (mAP, F1-score, and Recall values) are given in an easily understandable format. Furthermore, for each model, the status of the model is shown based on accuracy values using the visualization technique. If one of these three accuracy results under 80% (our threshold value for this study), color is changed for the related accuracy metric. Moreover, during the training process, training progress steps are given in a more understandable way using the text explanation technique, and at the end of the training operation, the meaning of accuracy values are interpreted by the application. The last trained model has activated automatically. On the other hand, users can activate one of the previous models from the list.

**Figure 5:**
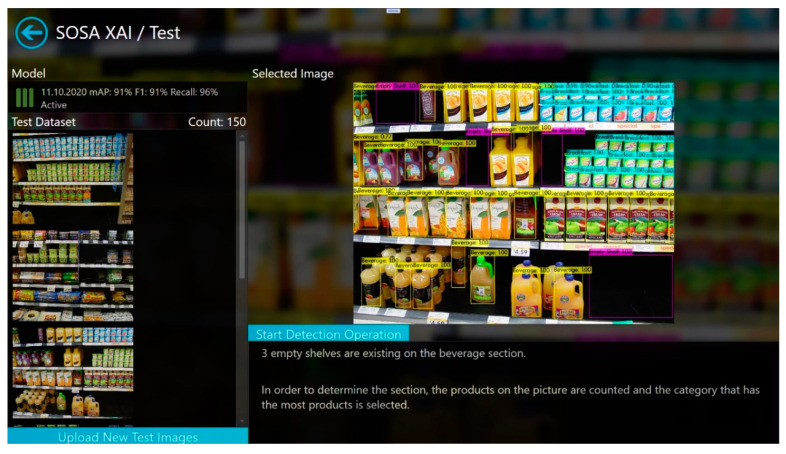
Test screen of SOSA XAI.

Thanks to our SOSA XAI application, the active trained model can be tested using a test screen. Test images are listed on the screen and also new test images can be uploaded. Accuracy values and creation date of the trained model are shown in a straightforward manner. For selected or uploaded test images, detection operation can be started and results are shown both visually and textually. In the textual explanation section, the decision of the model is given in an understandable way and explained how this decision is made. Figure 5 shows a screenshot of the object detection results. Besides, when some of the classes are detected with low accuracy, the application gives suggestions to extend the existing dataset to increase accuracy. For instance, when empty-shelf accuracy is less than 80%, the system suggests adding more images that contain more empty shelves from different perspectives and re-training the model. In the metrics screen of the software, the system shows sample images containing empty shelves from different perspectives, and users can expand the training set by adding new images among these samples. Moreover, the monitoring screen has also similar functionalities, but here, the user can design the test set, instead of the training set. From the monitoring screen, real-time results can be tracked the same as the test screen.

**Figure 6:**
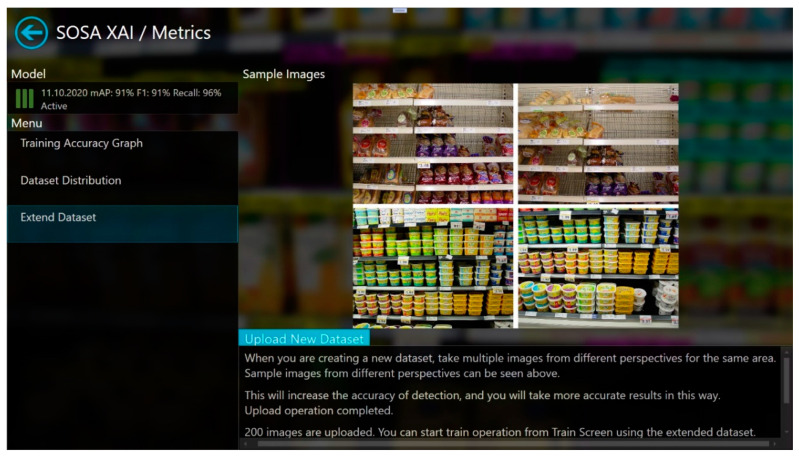
Metrics screen of SOSA XAI.

Finally, from the metrics screen (Figure 6), the training accuracy graph for the active trained model, and dataset distribution based on classes can be seen easily. In addition, the dataset can be extended using the “Extend Dataset” part with unlabeled images. Sample images are shown to give an idea of how to create a new dataset and suggestions are given as text for this purpose. After the upload operation is completed, the training process can be started from the training screen for the extended dataset. Text explanation, visualization, and explanation by example post-hoc techniques are used in this screen.

In a real-time application, shelf images can be taken approximately every hour during peak times and every three hours during non-peak times to be analyzed by the OSA system. Since this period is parametric in the developed system, it can be easily modified. In order to protect the privacy of customers, when an image is taken, an object detection approach [26] is firstly applied to check the presence of people on the image. If people are detected, this image is not processed by the system, and a new image is taken after a period of time until the image does not contain a person (i.e., shopper or employee). After this step, the trained model is used to classify the new image, and the image is stored in the system. When empty or almost-empty shelves are detected, a notification is sent to the responsible store clerk to check the related area. When a notification is received, a responsible clerk can put new products on the shelf to prevent customer and profit loss.

SOSA XAI gives an opportunity to understand, trust, and manage AI applications to increase OSA for users who are not AI experts and engineers in grocery stores. Results can be interpreted easily and the dataset can be extended with unlabeled images for requirements changes. Furthermore, the results of the previous models can be compared to the current one and the desired model can be selected and activated. It combines our proposed SOSA approach and XAI.

## 4. Experimental Studies and Results

In the experiments, the proposed approach SOSA was tested on a real-world dataset. A labeled dataset is needed for the test operation but the largest and well-known computer vision datasets do not provide annotation for grocery store products. In the literature, few datasets have been collected from grocery stores. On the other hand, all of these datasets have unlabeled images for the store products, and these datasets cannot be used directly without labeling some of them. 

We used the WebMarket dataset [27] in this study. The dataset contains 3153 unlabeled images that were taken from in front of shelves using three digital cameras that were standing from about 1 m away from shelves. The images were taken without any special illumination changes and without any viewpoint constraint but most of the images are frontal views. The images in the dataset were collected from 18 shelves in a retail store, each of length 30 m and each of which approximately has six levels. Each image generally covers an area of about 2 m in width and 1.5 m in height on shelves, including all the items within three or four shelf levels in range. The dataset contains images of 100 different items. The dataset also includes fine-grained product categories having minor variations in packages [28]. Since the model is trained on such data, it has the capability of dealing with packages. The format of each image is Jpeg and its resolution is either 2592 × 1944 or 2272 × 1704. High resolution holds sufficient information for each object appearing in the image, and thus, the trained model can deal with packages of multiple items and damaged packages, at least with lower accuracy. Until now, the dataset was used for object retrieval studies [29,30]. Namely, our study is the first study that uses the WebMarket dataset for monitoring on-shelf availability.

**Figure 7:**
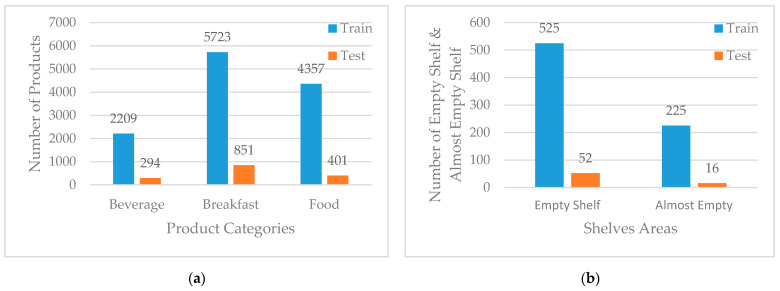
(**a**) Distribution of labeled products; (**b**) Distribution of labeled shelves’ areas.

In order to label the images, LabelImg [31] open-source tool was used and 300 images were labeled based on five classes. Three of them are for product categories that are “Beverage Products”, “Breakfast Products”, and “Food Products”, and two of them are for shelves’ areas that are “Empty Shelf” and “Almost Empty Shelf”. “Beverage Products” and “Breakfast Products” were selected from fast-moving consumer goods (FMCG) which are nondurable products, consumed at a fast pace. A total of 13,835 products were grouped under three categories, and 818 shelves’ areas grouped under two categories. The labeled dataset was divided into a training set (90%) and a test set (10%). The distribution of labeled products and shelves’ areas are shown in Figure 7.

**Figure 8:**
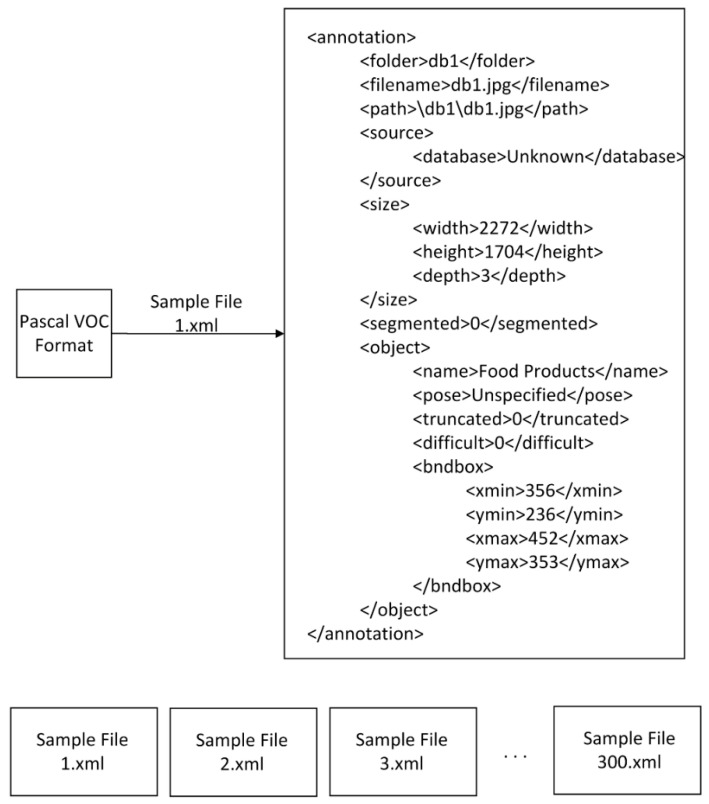
Structure of Pascal visual object classes (VOC) format for a sample file.

In this study, a file was created to store annotation info for each image, and Pascal visual object classes (VOC) was used as an annotation file, which contains all information about the images such as bounding box coordinates and class names. A sample Pascal VOC format is shown in Figure 8. Since it is in an XML file format, its format can be easily converted to other formats. Different object detection algorithms were used for different file formats to store labeling information of items on the images. Since products and shelf areas are annotated in a Pascal VOC format, we developed several conversion tools in C-Sharp to convert its format to the required formats.

**Figure 9:**
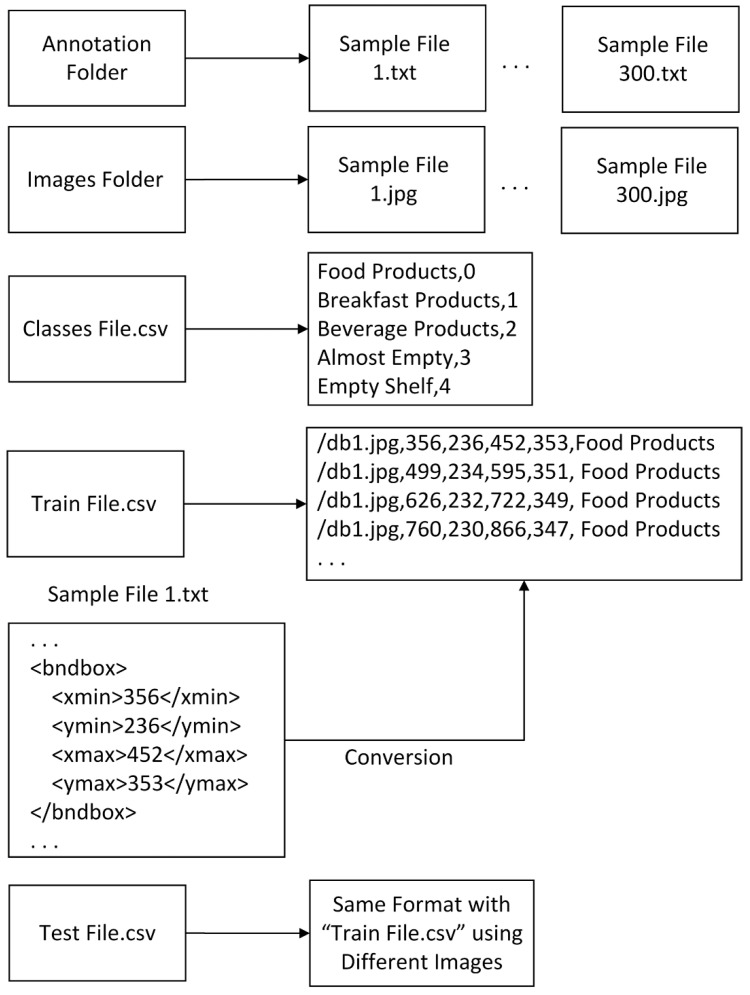
RetinaNet sample file structure and conversion details.

For RetinaNet, each file extension was converted from XML to TXT. Sample file structure and conversion details are shown in Figure 9. The class labels of the products were inserted in a CSV file. Finally, CSV files for the training and test sets were generated from the Pascal VOC file.

**Figure 10:**
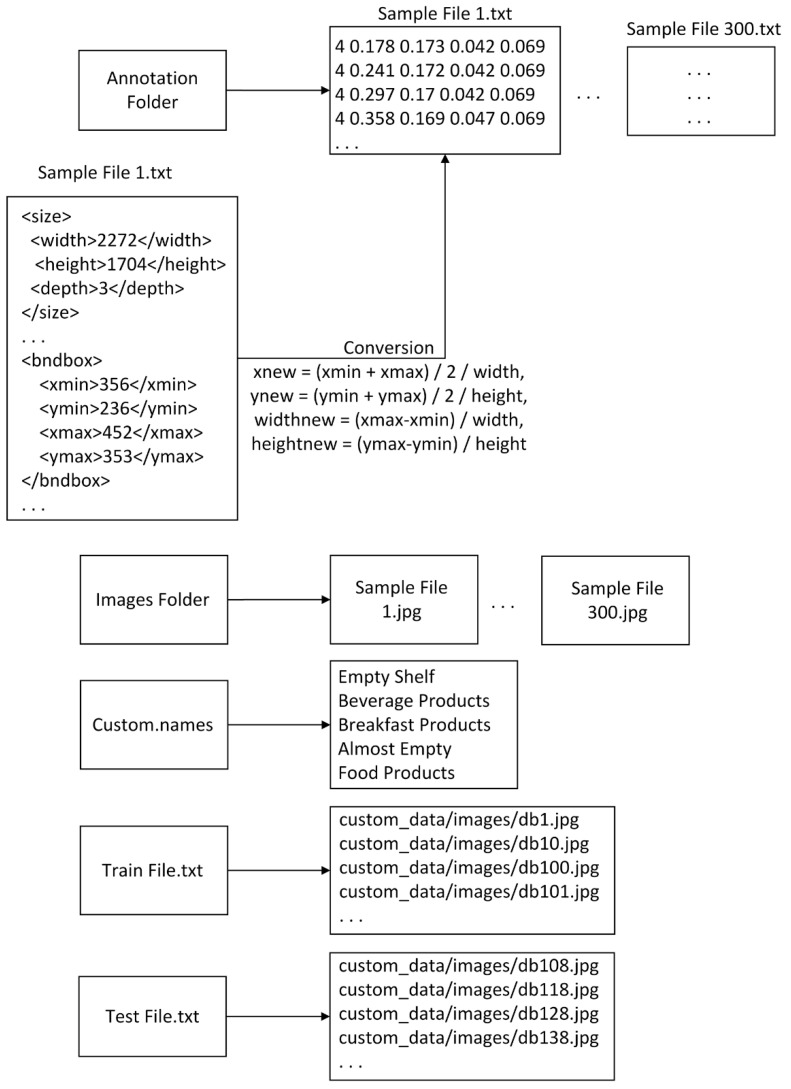
YOLOv3 and YOLOv4 sample file structure and conversion details.

For YOLOv3 and YOLOv4, each file extension was converted from XML to TXT, and conversion was applied from Pascal VOC to YOLO format. A sample file structure and conversion details are shown in Figure 10. The class labels of the products were inserted in a NAMES file. Finally, TXT files for the training and test sets were generated to store the locations of images.

For evaluating our proposed approach, first of all, a training process was started on the selected three one-stage detectors (RetinaNet, YOLOv3, and YOLOv4) using labeled images. The experimental environment was created on a computer that has the following specifications: Ubuntu 20.04 operating system, GeForce RTX 2080 TI GAMING X TRIO 11 GB graphical processing unit (GPU), AMD Ryzen 7 3700X 3.6 GHz/4.4 GHz processor, and 32 GB 3600 MHz DDR4 memory. The training operation is processed on the GPU for each detector.

The following open-source frameworks were used in this study: Keras-based framework for RetinaNet [32], Darknet-based framework for YOLOv3 [33], and YOLOv4 [34]. For each detector, the following parameters were determined: input image size as 512 × 512, learning rate as 0.001, and the number of iterations as 10,000. RetinaNet was trained using two different convolutional neural networks (CNN)-based backbones: ResNet50 and ResNet100. Darknet53, which is a new feature extraction network, was used as the backbone of YOLOv3. For YOLOv4, the CSPDarknet53 backbone was used. This backbone uses cross stage partial network (CSPNet) approach for partitioning and merging feature maps based on a cross-stage hierarchy [22]. Besides, for each backbone, pre-trained models were used, which are “model50.h5” and “model101.h5” for RetinaNet, “yolov3.weights” for YOLOv3, and “yolov4.conv.137” for YOLOv4.

At the end of the first training process, YOLOv4 with CSPDarknet53 had the highest success rates; 0.9187 for mAP, 0.9100 for F1-score, and 0.9600 for recall. These results were followed by RetinaNet with ResNet101 and ResNet50 backbones, and YOLOv3 with Darknet53 backbone. More details of the training results are shown in Table 2. From the results, it can be clearly seen that a significant performance improvement (>20%) was achieved by the proposed method on average. For example; YOLOv4 (0.9616) is significantly better than RetinaNet (0.7245) in the detection of breakfast products. YOLOv4 remarkably outperformed the rest for the “Empty Shelf” and “Almost Empty Shelf” classes with 0.9136 and 0.8125 of average precision values respectively.

In this study, we propose semi-supervised learning on OSA, named SOSA, for the first time to take the advantage of unlabeled data because labeling products on image data is an expensive, tedious, difficult, and time-consuming process. For this reason, products and shelf areas were labeled on a small number of images to compare our SOSA approach with the standard supervised OSA approach. The one-stage detector that had the highest success rate was selected with their trained model from the first training phase to use for the semi-supervised learning approach. The proposed SOSA method was evaluated on image datasets, with different ratios of labeled samples varying from 20% to 80%. The distribution of samples is shown in Table 3. For example, in the first experiment, 300 labeled images and 1200 unlabeled images were used to build a classifier. Firstly, unlabeled images were labeled using the selected one-stage detector with its model constructed in the previous training phase. In this way, a pseudo-labeled image dataset is obtained from an unlabeled image dataset. A total of 49,573 products were grouped under three categories and 2145 shelf areas were grouped under two categories for both labeled and pseudo-labeled images.

**Figure 11:**
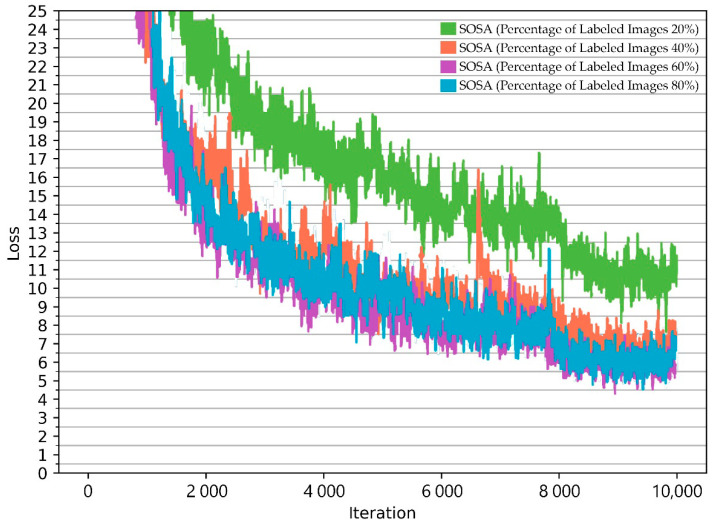
Comparison of loss value changes during the training phase.

Training processes were started for each dataset given in Table 3 to verify the effectiveness of the proposed SOSA method. Here, this experiment is performed to test the different ratios of labeled images, varying from 20% to 80% with an increment of 20%. The comparison of loss values obtained during the training phases is shown in Figure 11. From the figure, it can be seen that loss values decreased dramatically during the training phase after two thousand iterations. Very close loss values were obtained for different ratios. The results indicate that a small amount of labeled data is enough to receive similar loss values since the algorithm benefits from unlabeled data. From the results, it can be concluded that labeled data that contain shelf images taken from a grocery store can be extended with unlabeled images to achieve high accuracy.

**Figure 12:**
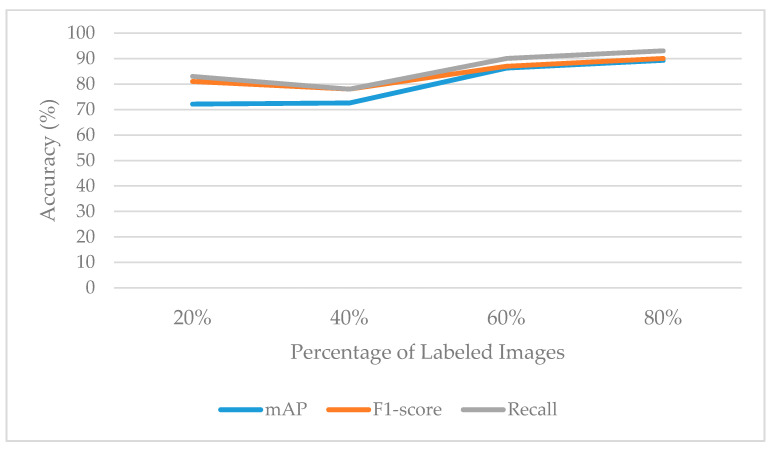
Success rates when different ratios of labeled data are considered.

As shown in Figure 12, satisfactory results were achieved by the proposed SOSA approach. When the percentage of labeled images was 20%, mAP, F1-score, and recall values were obtained as 0.7209, 0.8100, and 0.8300, respectively. When the percentage of labeled images was 40%, mAP was calculated as 0.7257, F1-score was achieved by 0.7800, and recall was measured as 0.7800. When using a 60–40% labeled-unlabeled ratio, mAP was 0.8622, F1-score was 0.8700, and recall was 0.9000. When the labeled image rate was 80%, mAP was 0.8927, F1-score was 0.9000, and recall was 0.9300. As has been observed in Figure 12, it is possible to provide good generalization ability for the OSA problem by applying a small number of labeled image data. It also can be seen from the results that an improvement could be expected from our method in circumstances where the ratio of labeled data grows. For example, after adding 20% labeled data, we found that the accuracy of the SOSA method increased from 0.7257 to 0.8622, with approximately 14% improvement. The results indicate that an initial amount of labeled ordinal data can be sufficient enough to learn the model, but additional labeled data can improve the classification performance.

**Figure 13:**
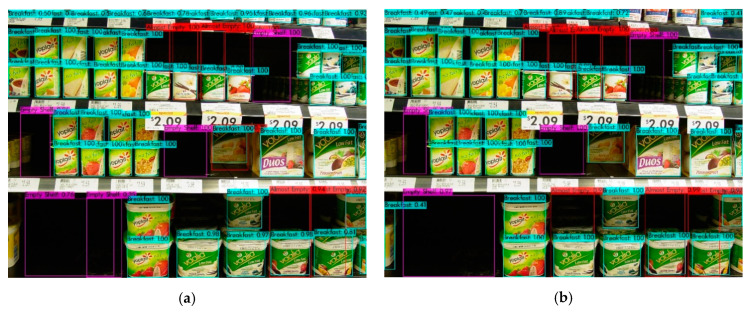

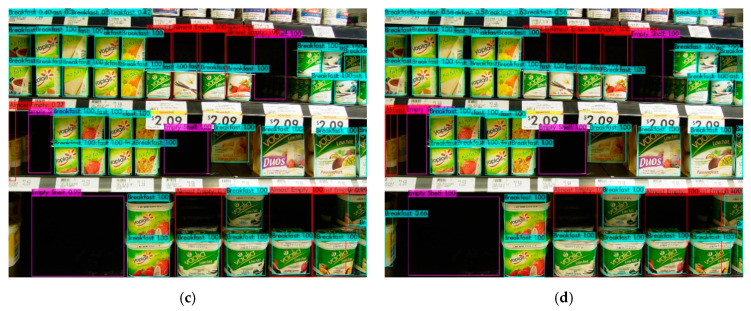
Sample object detection results of the breakfast section. It shows detection results using the model that was trained with (**a**) 20% of labeled images; (**b**) 40% of labeled images; (**c**) 60% of labeled images; and (**d**) 80% of labeled images.

The sample object detection results related to the breakfast section are shown in Figure 13. Each subfigure shows the results obtained by the models that were trained using labeled samples varying from 20% to 80%. While some almost-empty areas, which are highlighted in red color, could not be detected with a 20–80% labeled-unlabeled ratio, these areas could be detected with higher labeled data ratios. The detection accuracy of almost-empty areas was increased as the labeled data ratio was increased. Besides, from the SOSA XAI perspective, the results were interpreted in a more understandable way for users. SOSA XAI output message is as follows: “8 almost-empty and 4 empty shelves are existing on the breakfast section. In order to determine the section, the products on the picture are counted and the category that has the most products is selected”.

**Figure 14:**
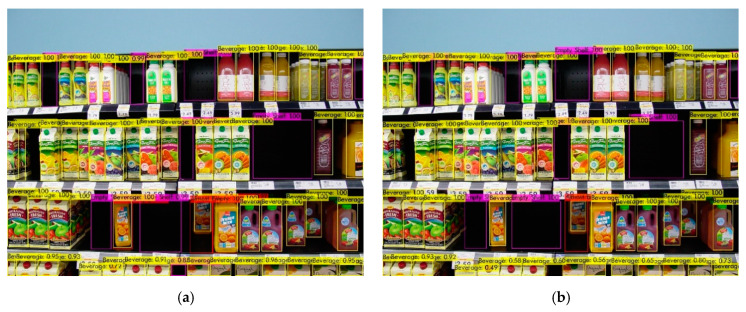

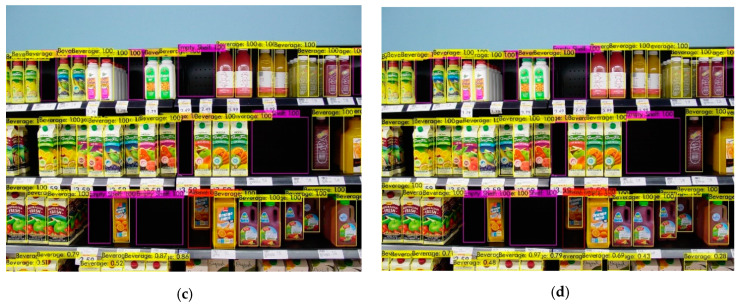
Sample object detection results of the beverage section. It shows detection results using the model that was trained with (**a**) 20% of labeled images; (**b**) 40% of labeled images; (**c**) 60% of labeled images; and (**d**) 80% of labeled images.

For the beverage section, the sample object detection results are shown in Figure 14. The sample shelf image has eight empty and one almost-empty shelves. All trained models detected empty shelves (highlighted in violet color) and almost-empty shelf (highlighted in red color) with high probabilities. For example, the model that was trained with 20% labeled images detected one of the empty shelves with 99% probability and the almost-empty shelf with 100% probability. The model that was trained with 80% labeled images detected one of the empty shelves with 100% probability and the almost empty shelf with 100% probability. Besides, all models detected beverage products with high probabilities.

**Figure 15:**
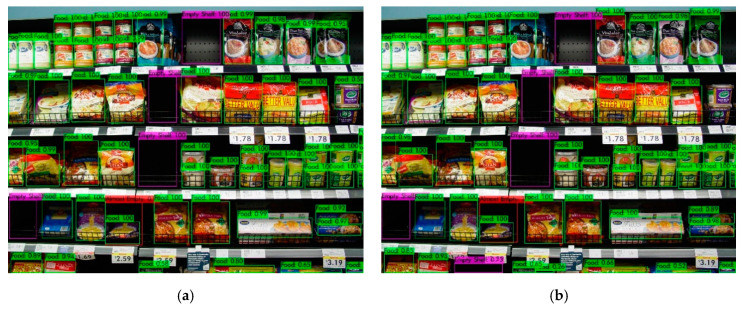

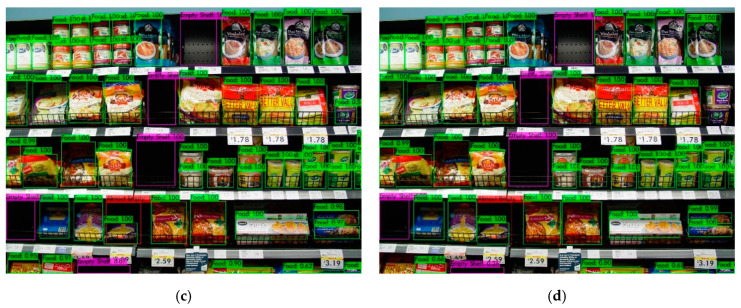
Sample object detection results of the rice and pasta section. It shows detection results using the model that was trained with (**a**) 20% of labeled images; (**b**) 40% of labeled images; (**c**) 60% of labeled images; and (**d**) 80% of labeled images.

In this study, we focused on detecting empty and almost-empty areas on the breakfast and the beverage sections. The images that do not include beverage and breakfast products are labeled as “Food Product”. The SOSA approach was also tested using shelf images that were taken from the food sections. Figure 15 shows the sample object detection results for the rice and pasta section. The results showed that products were correctly labeled as “Food Product” with high probabilities. Most of the images in the dataset are frontal views and products in front of the shelves are fundamentally analyzed to be detected in this study. When a product is on the backside of a shelf, the system generates an almost-empty shelf notification. However, if the depth of a shelf is too deep, the system may detect the products at the backside with low probability. In the cases of empty and almost-empty shelf notifications, a responsible clerk can put new items on the shelf to prevent customer and profit loss. It can also be noted that a package may contain multiple items but it may look like a single object from the outside, and in this case, the package is detected as a single object by the system, as expected. Besides, some products can be damaged. In this case, these products can also be detected by the system, but with lower probability due to the change in their appearance.

The comparison of the proposed SOSA approach with the existing approaches in terms of accuracy is shown in Table 4. The experimental results showed that the proposed approach outperformed the existing approaches (RetinaNet and YOLOv3) in terms of accuracy. The best performance was achieved for the breakfast products. When the percentage of labeled images varied between 80% and 20%, the AP values were obtained as 0.9467, 0.9626, 0.9253, and 0.8414, respectively. At most, all the algorithms had difficulty in distinguishing the “Almost Empty” class. Compared to the existing methods, approximately 25% improvement was achieved for the almost-empty class by the proposed method. Besides, this achievement of the SOSA method can be increased by extending the dataset with unlabeled images that contain more almost-empty areas. From the empty-shelf class perspective, satisfactory AP results (>0.8) were obtained by the proposed method. In addition, when AP results were evaluated based on product classes, it was seen that AP values increased compared to the previous methods on average. Based on the results, it can be concluded that the proposed SOSA method has good generalization ability in distinguishing all the classes, so it can be effectively used to recognize them well. 

## 5. Discussion

Providing high on-shelf availability is a key factor to increase profits in grocery stores. For this purpose, this study is the first attempt to combine two concepts “semi-supervised learning” and “on-shelf availability” (SOSA). The proposed SOSA method aims to decrease out-of-stock by automatically detecting and classifying products using shelf images based on the semi-supervised learning principle. The main purposes of the SOSA method are to decrease the need for manual image labeling, to obtain satisfactory results using a small amount of manually labeled images, and to provide additional knowledge present in unlabeled image data.

SOSA builds a classification model that allows the detection of “Product”, “Empty Shelf”, and “Almost-Empty Shelf” classes by using both labeled and unlabeled image data. When building the model, three different deep learning techniques are used: RetinaNet, YOLOv3, andYOLOv4. This study shows that the proposed approach improves accuracy when monitoring OSA.

In the experimental studies, the effectiveness of the proposed SOSA method was demonstrated on a real-world image dataset, with different ratios of labeled samples varying from 20% to 80%. As can be seen from Table 4, the proposed SOSA approach outperformed the previous approaches on average. For instance, according to the experimental results, 15–18% improvement is achieved on average accuracy.

When developing an OSA system, some challenges could be encountered and overcome. The first one is that some products can lie on the shelf or their packages can be damaged. In these cases, these kinds of products can be detected with low probability due to the change in their appearance. To increase detection probability, the images containing this type of product can be included in the training set. In this way, the model can learn from these kinds of objects, and therefore these products can be detected with higher accuracies. Another difficulty is that some objects can stand in front of the shelf and they can prevent detecting products on the shelf. These objects can be shoppers, employees, grocery store trolleys, or something else. In this study, we implemented a solution to check the presence of people on the image. If a person is detected, this image is discarded by the system, and then a new image is taken after a period of time. This issue is also important to protect the privacy of customers. Similar solutions can also be implemented for other objects. 

The SOSA method is powered by the SOSA XAI application. SOSA XAI was developed to provide transparency and understandability of the OSA model for the users. Thanks to this tool, detection results can be interpreted more easily. Furthermore, it provides insight into what the OSA model is paying attention to. In addition, it provides evidence for the results, and validity of the process. Moreover, the training set can be enhanced in a proper manner to adopt the application for changes without an expert in the AI field. 

The main findings of this study can be listed as follows:

(1) It was observed that “semi-supervised learning” provides many advantages for monitoring OSA, including improving efficiency, reducing labeling cost, providing additional knowledge present in unlabeled data, and increasing the applicability of machine learning in the retail sector.
(2) The combination of three deep learning techniques (RetinaNet, YOLOv3, YOLOv4) improves accuracy when monitoring OSA.
(3) Explainable AI is a powerful tool in monitoring OSA since it provides users with an explanation of individual decisions and enables users to manage, understand, and trust the OSA model.
(4) The proposed SOSA method has the potential to expand the application of machine learning in grocery stores, thanks to its advantages.

## 6. Conclusions and Future Work

Providing high on-shelf availability is a key factor to increase profits in grocery stores. For this purpose, the traditional OSA applications use the labeled image data when building a classifier. However, a large amount of data generated in real-life is unlabeled, and manually labeling products on the images is an expensive process. To overcome this problem, this paper proposes a new approach, called SOSA, which combines two concepts “semi-supervised learning” and “on-shelf availability” for the first time. The proposed SOSA method addresses the problem by automatically allowing the model to integrate the available unlabeled image data with very little cost. Our proposed method detects empty and almost empty shelves based on each product category using a small amount of labeled data and a large amount of unlabeled data.

It is the first time that YOLOv4 object detection architecture is used for monitoring OSA. This study is also original in that it compares three deep learning approaches (RetinaNet, YOLOv3, YOLOv4) for monitoring OSA in the retail sector.

The experiments were conducted on image datasets with different ratios of labeled samples varying from 20% to 80%. The experimental results show that the proposed approach outperforms the existing approaches (RetinaNet and YOLOv3) in terms of accuracy.

Moreover, this paper presents the first demonstration of explainable artificial intelligence (XAI) on OSA, called SOSA XAI. Thanks to the SOSA XAI, users can understand, trust, and manage the proposed deep learning model and modify the dataset to adapt the deep learning model for changes.

Currently, the developed SOSA XAI software application uses rule-based interpretation to explain the outputs of the proposed approach. Instead of rule-based interpretation, a deep learning-based interpretation can be developed in the future. Furthermore, augmented reality technology can be implemented to render some virtual objects related to the products detected on the shelves. Moreover, more than three one-stage detectors can be integrated into the SOSA approach as future work.

## Figures and Tables

**Table 1:** Comparison of the proposed Semi-Supervised Learning on on-shelf availability (SOSA) approach and the previous approaches.

Reference No	Year	Object Detection			Learning		XAI	Methods
		Traditional Detection	Deep Learning		Supervised Learning	Semi-Supervised Learning			
			Two-Stage	One-Stage				
[5]	2015	✔			✔		✕	SURF	
[9]	2015	✔			✔		✕	Image Processing	
[10]	2015	✔			✔		✕	Image Processing	
[12]	2015	✔			✔		✕	k-d Tree, RANSAC	
[16]	2015	✔			✔		✕	Hausdorff Map, Euclidean Distance, Binary Distance Map	
[11]	2016	✔			✔		✕	AKAZE Feature Detector, Cascade Classifier	
[6]	2018		✔		✔		✕	CaffeNet-based Network, CIFAR-10-based Network	
[13]	2019		✔		✔		✕	CaffeNet-based Network, CIFAR-10-based Network, Hungarian	
Proposed Method				✔		✔	✔	RetinaNet, YOLOv3, YOLOv4	

**Table 2:** Comparison of success rates of the methods on the labeled images for three different one-stage detectors with four different backbones.

Classes	RetinaNet (Backbone: ResNet50)	RetinaNet (Backbone: ResNet101)	YOLOv3 (Backbone: Darknet53)	YOLOv4 (Backbone: CSPDarkNet53)
	AP			
Beverage Product	0.9469	0.9625	0.8636	0.9808
Breakfast Product	0.6975	0.7245	0.8003	0.9616
Food Product	0.8886	0.8697	0.6321	0.9252
Empty Shelf	0.8481	0.8387	0.8189	0.9136
Almost Empty Shelf	0.2386	0.1561	0.5884	0.8125
mAP	0.7239	0.7103	0.7406	0.9187
F1-score	0.7333	0.7430	0.6600	0.9100
Recall	0.8105	0.8228	0.6600	0.9600

**Table 3:** Distribution of labeled samples on image datasets.

Dataset ID	Percentage of Labeled Images	Number of Labeled Images	Number of Unlabeled Images	Number of Total Images
D1	20%	300	1200	1500
D2	40%	300	450	750
D3	60%	300	200	500
D4	80%	300	75	375

**Table 4:** Comparison of accuracies between SOSA and existing approaches.

Classes	RetinaNet (Backbone: ResNet50)	RetinaNet (Backbone: ResNet101)	YOLOv3 (Backbone: Darknet53)	SOSA (80% Labeled Images)	SOSA (60% Labeled Images)	SOSA (40% Labeled Images)	SOSA (20% Labeled Images)
	AP						
Beverage Product	0.9469	0.9625	0.8636	0.9586	0.8797	0.7477	0.8224
Breakfast Product	0.6975	0.7245	0.8003	0.9467	0.9626	0.9253	0.8414
Food Product	0.8886	0.8697	0.6321	0.9303	0.8680	0.7308	0.8718
Empty Shelf	0.8481	0.8387	0.8189	0.8410	0.8736	0.7601	0.7216
Almost Empty Shelf	0.2386	0.1561	0.5884	0.7866	0.7269	0.4646	0.3471
mAP	0.7239	0.7103	0.7406	0.8927	0.8622	0.7257	0.7209
F1-score	0.7333	0.7430	0.6600	0.9000	0.8700	0.7800	0.8100
Recall	0.8105	0.8228	0.6600	0.9300	0.9000	0.7800	0.8300

## Data Availability

Publicly available dataset was used in this study. This data can be found here: http://yuhang.rsise.anu.edu.au/.
